# Texture Classification by Texton: Statistical versus Binary

**DOI:** 10.1371/journal.pone.0088073

**Published:** 2014-02-10

**Authors:** Zhenhua Guo, Zhongcheng Zhang, Xiu Li, Qin Li, Jane You

**Affiliations:** 1 Shenzhen Key Laboratory of Broadband Network & Multimedia, Graduate School at Shenzhen, Tsinghua University, Shenzhen, China; 2 Graduate School at Shenzhen, Tsinghua University, Shenzhen, China; 3 College of Software, Shenzhen Institute of Information Technology, Shenzhen, China; 4 Department of Computing, The Hong Kong Polytechnic University, Hong Kong, China; University of Campinas, Brazil

## Abstract

Using statistical textons for texture classification has shown great success recently. The maximal response 8 (Statistical_MR8), image patch (Statistical_Joint) and locally invariant fractal (Statistical_Fractal) are typical statistical texton algorithms and state-of-the-art texture classification methods. However, there are two limitations when using these methods. First, it needs a training stage to build a texton library, thus the recognition accuracy will be highly depended on the training samples; second, during feature extraction, local feature is assigned to a texton by searching for the nearest texton in the whole library, which is time consuming when the library size is big and the dimension of feature is high. To address the above two issues, in this paper, three binary texton counterpart methods were proposed, Binary_MR8, Binary_Joint, and Binary_Fractal. These methods do not require any training step but encode local feature into binary representation directly. The experimental results on the CUReT, UIUC and KTH-TIPS databases show that binary texton could get sound results with fast feature extraction, especially when the image size is not big and the quality of image is not poor.

## Introduction

Texture analysis is an active and fundamental research topic in the fields of computer vision and pattern recognition. Generally speaking, there are four basic problems in texture analysis: classifying images based on texture content; segmenting an image into regions of homogeneous texture; synthesizing textures for graphics applications; and establishing shape information from texture cues [1]. Texture classification has been widely studied because of many potential applications, including fabrics inspection [2], remote sensing [3], and medical image analysis [4].

Early texture classification methods focus on the statistical analysis of texture images. The representative ones include the co-occurrence matrix method [5] and the filtering based method [6]. These methods could achieve good classification results if the training and testing samples are captured by similar orientations. To address the rotation invariance issue, some model-based methods were proposed, such as circular autoregressive model [7], multiresolution autoregressive model [8], hidden Markov model [9], and Gaussian Markov random field [10]. Recently, scale and affine invariance receive extensive attention, and some algorithms were developed to address this issue, such as fractal transform [11], and local phase information [12].

In fact, classifying texture images taken under arbitrary viewing and illumination conditions is a difficult task. The method of statistically representing image local features has achieved great success for this problem [13], two paradigms for image representation were proposed, Signature (representative descriptors of an image [13]) and Statistical Texton (“the putative units of pre-attentive human texture perception” [30] which does not have a specific definition.). In the former paradigm, an image is represented by signatures which are adaptively extracted from each image [14], and Earth mover's distance [15] is utilized to compare different images; in the latter paradigm, an image is modeled by feature texton histogram over a dictionary of textons [16]-[22], and histogram dissimilarity (usually chi-square) is used for histogram comparison [23]–[24].

Statistical texton based methods are simple to implement and could achieve good performance on texture image classification [16]–[22]. However, these methods suffer two disadvantages. First, it requires an offline step to learn a texton dictionary from training samples, thus the recognition accuracy is related with the training samples; second, to build the histogram, one needs to search the nearest texton from the dictionary for each pixel. This step is time consuming especially when the dimension of feature is high and the size of dictionary is large.

Local binary pattern (LBP) is a simple and efficient operator which labels the pixels of an image by thresholding the neighborhood of each pixel and considers the results as a binary number [25], and it is not influenced by the above mentioned issues. Inspired by the idea of LBP, in this paper three binary texton methods, Binary_MR8, Binary_Joint, and Binary_Fractal, were proposed. These methods do not require learning and they are fast for feature extraction. They could be regarded as the counterpart of the three state-of-the-art statistical texton methods, Statistical_MR8 [16]-[17], Statistical_Joint [18]-[19] and Statistical_Fractal [20]. Our previous work showed that Binary_MR8 could get better accuracy than Statistical_MR8 on CUReT database [26]. This paper extended previous work by proposing two new binary texton methods, Binary_Joint and Binary_Fractal, and by doing more comprehensive experiments on three databases, CUReT database [27], UIUC database [14] and KTH-TIPS database [28].

The rest of the paper is organized as follows. Section 2 introduces three statistical texton methods. Section 3 shows the three proposed binary texton methods and dissimilarity metric. Section 4 reports the experimental results on three texture databases. Section 5 gives the conclusion and provides a suggestion for future work.

## Review of Statistical Texton Methods

### 1. Review of Statistical_MR8 [16]–[17]

**Figure 1:**
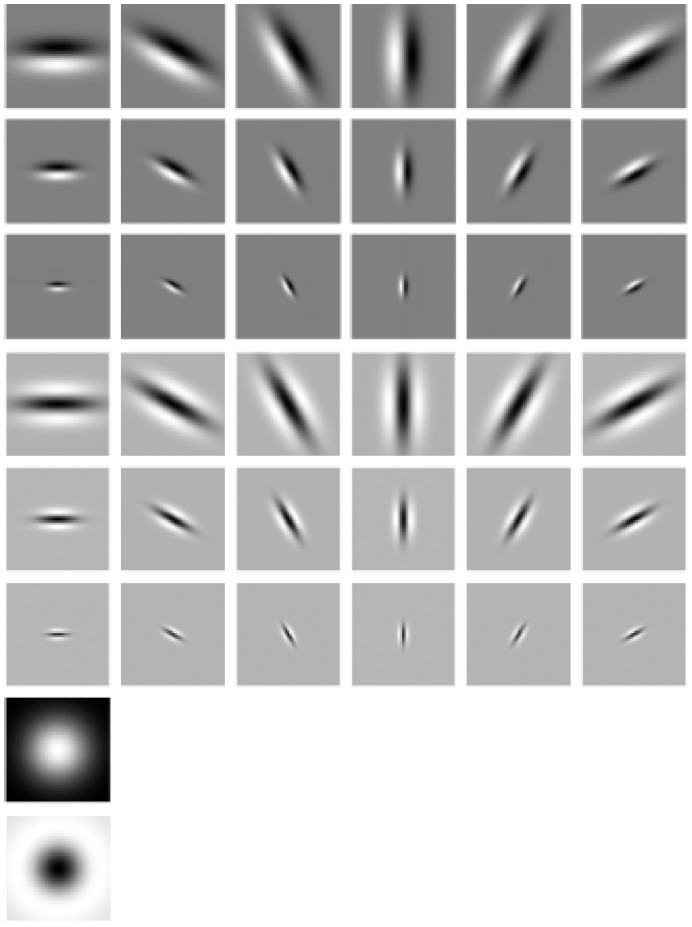
The Statistical_MR8 filter bank consists of a series of anisotropic filters (an edge and a bar filter at 6 orientations and 3 scales), and 2 rotationally symmetric ones (a Gaussian and a Laplacian of Gaussian) [16]–[17].

The Statistical_MR8 filter bank consists of 38 filters, which are shown in Fig.1. To achieve rotation invariance, the filters are implemented at multiple orientations and on multiple scales. On each scale only the maximal response among the different orientations is kept. The final response at each pixel is an 8-dimension feature vector (3 scales for the edge and bar filters, plus 2 isotropic filters).

During dictionary learning, a selection of *n* images is chosen for each class of texture, and the filter responses to all these images are aggregated, then *c* texton cluster centres are computed using the standard K-Means algorithm [29]. The learnt textons for each texture are then collected into a single dictionary (n*c). For a given image, after getting 8-dimension feature vector for one pixel, the feature is searched in the dictionary to find the closest one and label the pixel with that texton. Finally, an appearance frequency of all textons in the whole dictionary is built as the histogram feature for the image.

### 2. Review of Statistical_Fractal [20]

**Figure 2:**
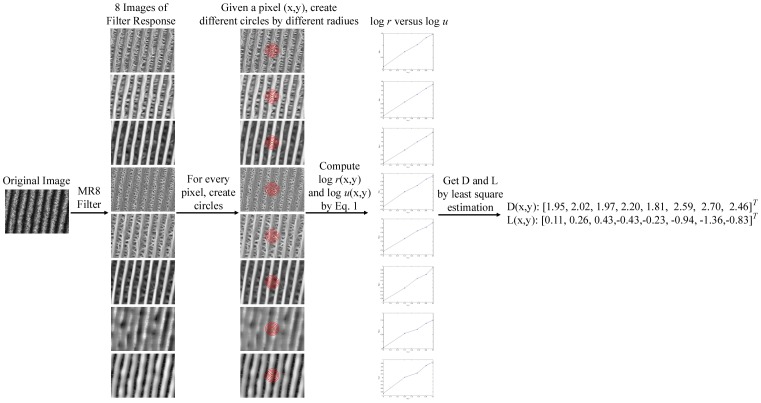
Illustration of computation for Statistical_Fractal [20].

To address the scale and affine issue, fractal feature was proposed based on Statistical_MR8 filter banks [20]. Given an image point (*x*, *y*), after getting 8-dimensional filter response by Statistical_MR8 filter banks, 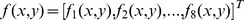, fractal is computed by an assumption: given a suitable measure 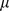, the “size” of local point sets in textured images follows a local power law. 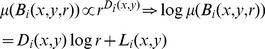
(1)where 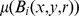
is the sum of all pixel filter responses of the 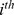 dimension (*i* = 1,2, …,8) that lies within a closed disk *B* of radius *r* centered at an image point (*x*, *y*), 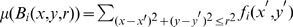. 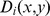 (slope of 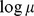
versus 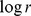) and 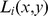 (intercept of 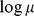 versus 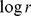) are computed by least square estimation. The former is the local fractal dimension and is invariant to scales changes, while the latter is the local fractal length and is rotation invariant only [20]. Fig. 2 illustrates an example to compute *D* and *L*. Two new 8-dimension features, *D*(*x*, *y*) and *L*(*x*, *y*) for each pixel is computed, after that the same dictionary learning and feature extraction procedure as Statistical_MR8 is used. In the following figure, Statistical_Fractal_D and Statistical_Fractal_L represent the texton histogram of *D* and *L*, respectively.

### 3. Review of Statistical_Joint [18]-[19]

**Figure 3:**
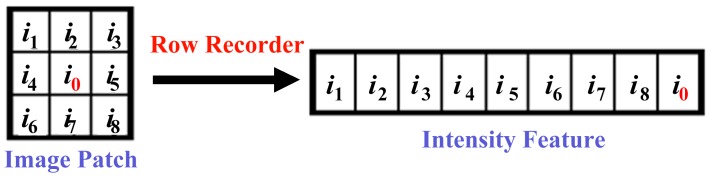
Illustration of texton feature for Statistical_Joint [18]–[19]. A 3*3 image patch is converted to a 1*9 texton through recording intensity values row by row.

Instead of applying filters on gray level images, Statistical_Joint is proposed to use multi-dimension intensity value (gray value) for each texton [18]–[19]. For a point *x*, a r*r rectangle around *x* is selected and the intensity of the rectangle is used to represent the texton feature for the point. Fig. 3 shows an example for *r* = 3.

## Proposed Binary Texton Methods and Dissimilarity Metric

**Figure 4:**
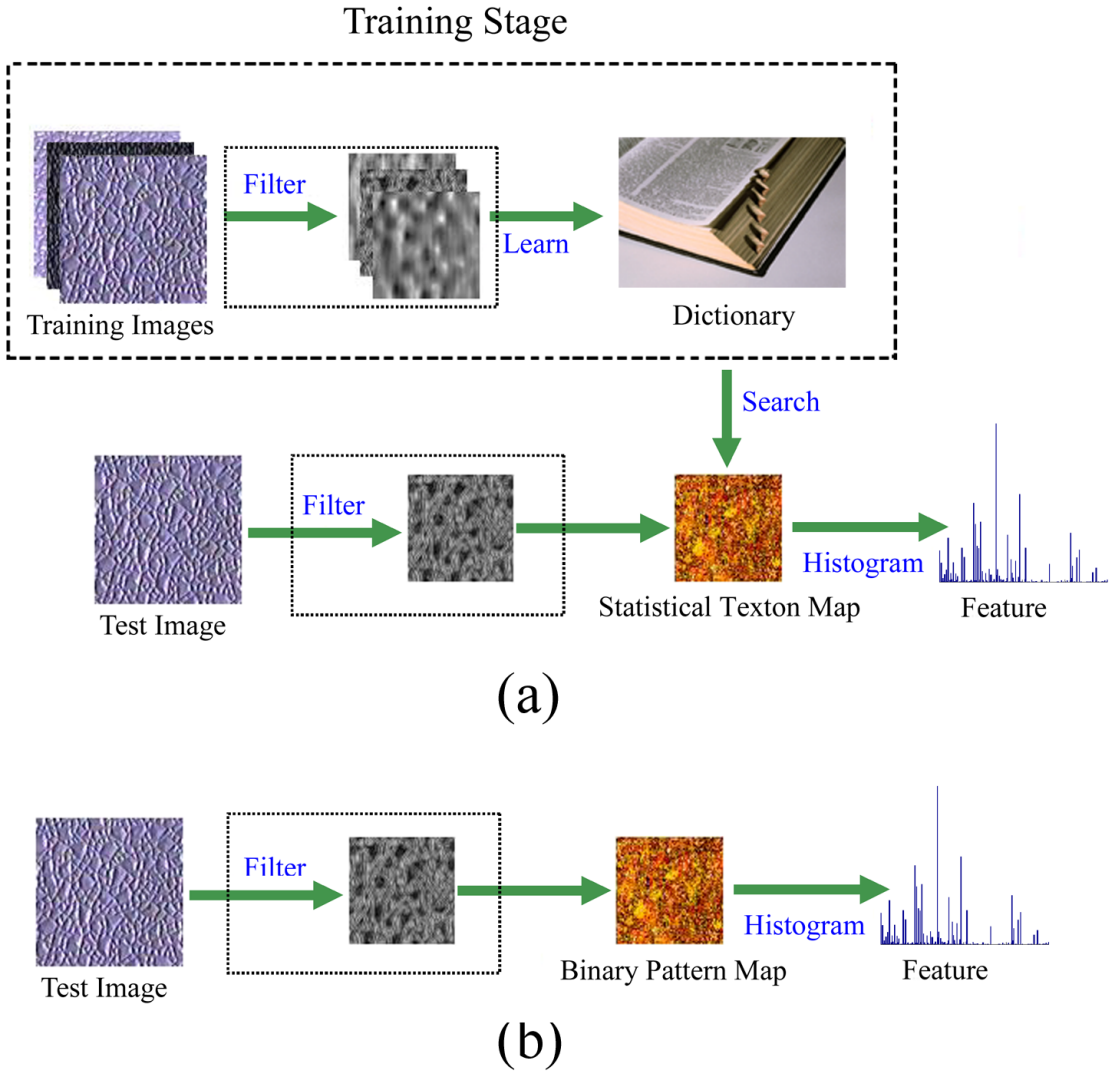
Major framework of texton feature extraction. **a) statistical texton; b) binary texton.**

Fig. 4 shows an overview of the statistical texon and binary texton methods. As illustrated in Fig. 4, binary texton is extracted from intensity value or filter response directly, it does not need any training stage and is fast to build the feature map.

### 1. Binary_MR8

**Figure 5:**
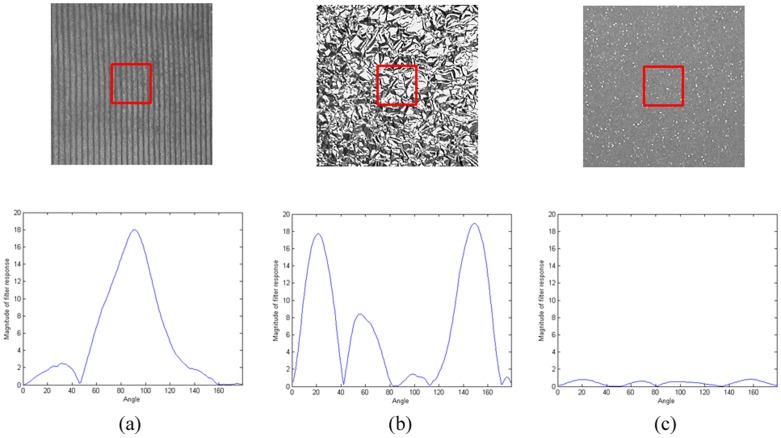
The top row shows 3 texture images. The central image patch (highlighted by red rectangle) is matched with an edge filter at all orientations. The magnitude (absolute value) of the filter response versus the orientation is plotted in the bottom row.

As shown in Fig.5, some local regions may have multiple dominant orientations. The magnitude of the filter response at each angle could be treated as a confidence measurement in the feature occurring at that orientation [17]. It is intuitive to define a binary texton for multiple orientations as:
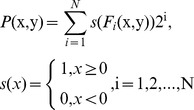
(2)

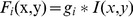
(3)where 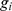 is the filter as shown in Fig. 1, * is the convolution operator, and *I* is the input image.

However, the feature length of Eq. (2) is too long, it has 38 bits and is not rotation invariant. Thus, the 38 bits is divided into 8 rows based on the filters shown in Fig. 1, for each row, a rotation invariant sub-texton is defined through one scale of filter(s). For the last two rows, as there is only 1-bit string, it is in nature rotation invariant. For the first six rows, the output is 6-bits binary string, a rotation invariant sub-texton designed based on the idea of “rotation invariant uniform” [25] is defined:
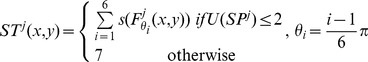
(4)

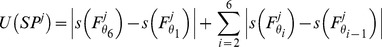
(5)where *j* is the index of row, and 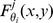 is the convolution output by the filter in the *j* ^th^ row and *i* ^th^ column as shown in Fig. 1. By definition, exactly 7 “rotation invariant uniform” binary patterns (“000000”, “000001”, “000011”, “000111”, “001111”, “011111”, “111111”) can occur in a circularly symmetric neighbor set of 6 binary bits, while the remaining (non-uniform) are grouped into a “miscellaneous” label (7). Thus, totally, there are 8 distinct output values for 6-bit binary string.

**Figure 6:**
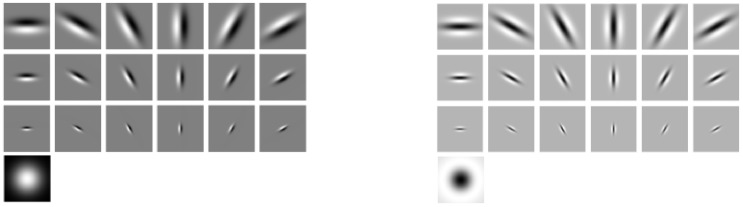
The Statistical_MR8 filter banks are divided equally into two groups.

**Figure 7:**
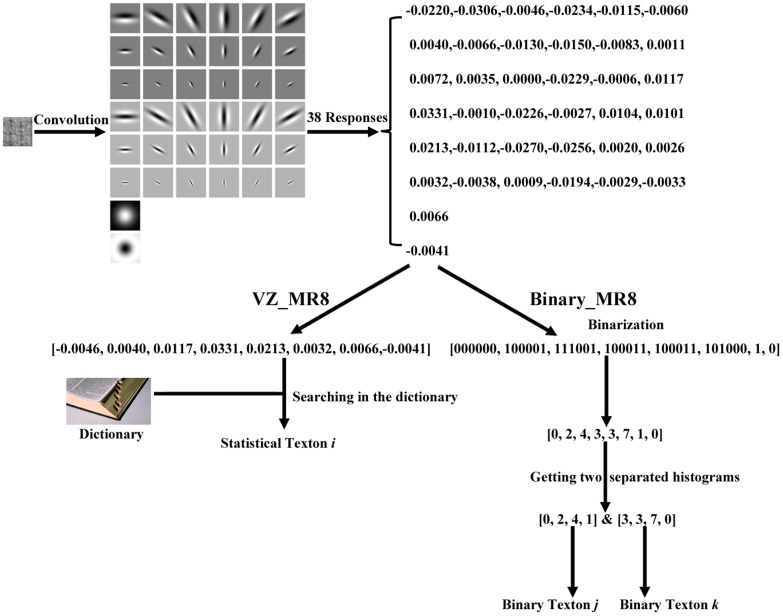
An illustration example of Statistical_MR8 and Binary_MR8.

The filtering output at each position is a 8-dimensional vector, and there are 1,048,576 (8*8*8*8*8*8*2*2) kinds of patterns in total. Such a dimension is too large to build histogram and it will bring a computation issue. To reduce the feature size, we empirically divide the 38 filters into 2 groups as shown in Fig. 6. Thus for each image, only two 4-dimensional histograms need to be built and then the 2 histograms are concatenated. The final histogram size is reduced to 2,048 (8*8*8*2*2). Fig. 7 shows an example to illustrate the difference between Statistical_MR8 and Binary_MR8.

### 2. Binary_Fractal

In Statistical_Fractal, only selective fractal values are left. As shown in Section 3.1, the texture image is complex and may contain multiple orientations at a local region. Similar to Binary_MR8, all fractal values are binarized and kept for feature extraction in Binary_Fractal.

After getting 38 filtered images, 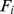
(*i* = 1, 2, …, 38), by Statistical_MR8 filter banks, 38 images of local fractal dimensions (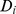) and 38 images of local fractal lengths (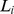) are gotten by Eq. (1) respectively. Unlike Binary_MR8, the local fractal values are all positive values, thus “0” which is used in Binary_MR8 could not be used as the threshold to get binary results. Instead, median value of each fractal image is selected as the threshold to get binary output:
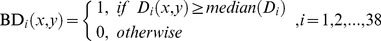
(6)




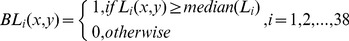
(7)Where 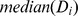 and 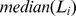 are median value of the whole image of 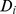 and 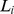 respectively.

Then, similar to Binary_MR8, for each position, the 38 binary bits of 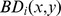
and 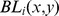
are divided into 8 rows respectively and for each row a rotation invariant sub-texton is defined. Finally, as discussed in Section 3.1, the 8 rows are divided into 2 groups to reduce feature size and a feature histogram with size 2,048 (8*8*8*2*2) is built for Binary_Fractal_D and Binary_Fractal_L respectively.

### 3. Binary_Joint

Although Statistical_Joint could get good results on texture classification, it is too slow for some real time applications. Taking 7*7 patch based Statistical_Joint as an example, it is time consuming to search 49-dimension statistical textons especially for a large size image [19]. Inspired by the idea of LBP, a binary counterpart of local patch is proposed.

As 7*7 patch based Statistical_Joint could get good results in most of applications [18]–[19], the Binary_Joint is defined on a 7*7 patch only. First, taking a position (*x*, *y*) in the original image as the central point, a 7*7 rectangle is selected from the original image. 49 binary bits are computed:
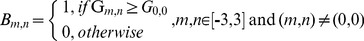
(8)

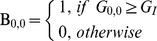
(9)where 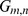 is the gray value at coordinate (*m*, *n*), and 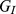 is the average intensity of the whole image.

**Figure 8:**
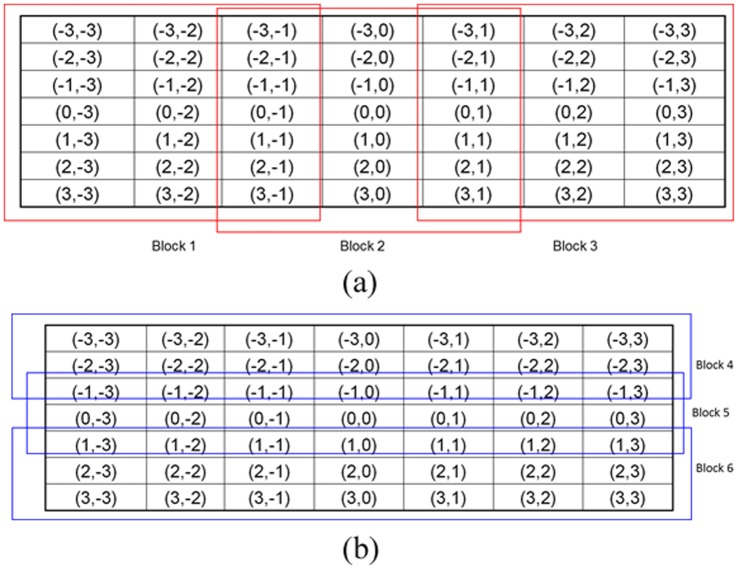
A 7*7 patch is divided into 6 blocks.

As 49 binary bits are too long for feature extraction, the 7*7 binary rectangle is divided into 6 blocks as shown in Fig. 8.

Then for each block, a sub-histogram is built. For blocks 1, 2, and 3, the 7 bits of the central column form a sub-texton:
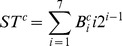
(10)Where 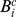
is the *i* ^th^ bit of the central column. There are 128 possible values for 7 bits. To reduce the feature size, the idea of “uniform” from LBP [25] is used to reduce the number of sub-textons. A sub-texton is labeled to “non-uniform”, if 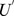, the number of bitwise 0/1 changes, is bigger than 2. 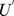
is defined as:
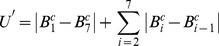
(11)Thus, the total number of possible sub-textons of the central column is 45. Table 1 lists the 45 kinds of sub-textons with their labels.

**Table 1:** 45 kinds of sub-textons with 7 bits and their labels.

“0000001” (Label: 0)	“0000010” (Label: 1)	“0000100” (Label: 2)	“0001000” (Label: 3)	“0010000” (Label: 4)	“0100000” (Label: 5)	“10000000” (Label: 6)
“0000011” (Label: 7)	“0000110” (Label: 8)	“0001100” (Label: 9)	“0011000” (Label: 10)	“0110000” (Label: 11)	“1100000” (Label: 12)	“1000001” (Label: 13)
“0000111” (Label: 14)	“0001110” (Label: 15)	“0011100” (Label: 16)	“0111000” (Label: 17)	“1110000” (Label: 18)	“1100001” (Label: 19)	“1000011” (Label: 20)
“0001111” (Label: 21)	“0011110” (Label: 22)	“0111100” (Label: 23)	“1111000” (Label: 24)	“1110001” (Label: 25)	“1100011” (Label: 26)	“1000111” (Label: 27)
“0011111” (Label: 28)	“0111110” (Label: 29)	“1111100” (Label: 30)	“1111001” (Label: 31)	“1110011” (Label: 32)	“1100111” (Label: 33)	“1001111” (Label: 34)
“0111111” (Label: 35)	“1111110” (Label: 36)	“1111101” (Label: 37)	“1111011” (Label: 38)	“1110111” (Label: 39)	“1101111” (Label: 40)	“1011111” (Label: 41)
“0000000” (Label: 41)	“1111111” (Label: 43)	“non-uniform” (Label: 44)				

For the left and right columns, the number of 1-bits is compared with the central column. There are three possible values. 
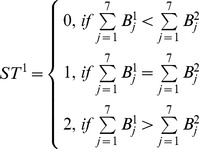
(12)

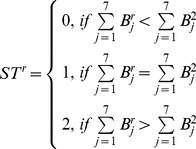
(13)Where 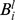
and 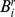
is the *i* ^th^ bit of the right and left columns, respectively. A sub-histogram based on [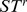, 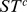, 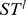] is built for the whole image. Thus, for blocks 1, 2, and 3, the feature size of a sub-histogram is 405 (45*3*3).

**Figure 9:**
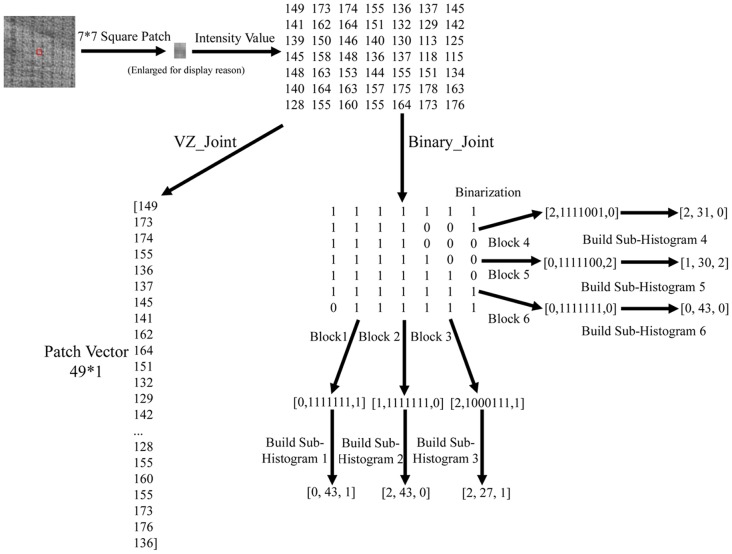
An illustration example of Statistical_Joint and Binary_Joint.

Similarly, for blocks 4, 5, and 6, a sub-texton is based on the central row, and the number of 1-bit is compared with the up and down rows. A sub-histogram with size of 405 is extracted for each block. Finally, six sub-histograms are concatenated and a feature histogram with 2,430 (405*6) bins is extracted for each image. Fig. 9 shows an example to illustrate the difference between Statistical_Joint and Binary_Joint.

### 4. Dissimilarity Metric

The dissimilarity of sample and model histograms is a test of goodness-of-fit, which could be measured with a nonparametric statistic test. There are many metrics to evaluate the goodness between two histograms, such as histogram intersection, log-likelihood ratio, and chi-square statistic [25]. In this study, a test sample *T* is assigned to the class of model *L* that minimizes the chi-square distance:
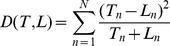
(14)where *N* is the number of bins, and 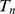 and 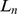 are the values of the sample and model image at the *n* ^th^ bin, respectively. In this paper, the nearest neighborhood classifier with chi-square distance is used to measure the dissimilarity between two histograms, because it is equivalent to the optimal Bayesian classification [23], and good performance for texture classification can be achieved [24].

## Experimental Results and Discussion

To evaluate the effectiveness of the proposed methods, we carried out a series of experiments on three large and comprehensive texture databases: the Columbia-Utrecht Reflection and Texture (CUReT) database, which contains 61 classes of real-world textures, each imaged under different combinations of illumination and viewing angle [27], University of Illinois at Urbana-Champaign (UIUC) database [14], which includes 25 classes and 40 images per class collected under significant viewpoint variations, and Kungliga Tekniska högskolan (Swedish) -Textures under varying Illumination, Pose and Scale (KTH-TIPS) database [28], which include contains 10 classes and 81 images per class imaged under different scales, different poses and different illumination conditions.

Except Binary_Fractal, the image sample was normalized to have an average intensity of 0 and a standard deviation of 1 [16]–[20] for different methods. This is to remove global intensity and contrast [16]–[20]. For Binary_Fractal, the image sample was normalized to have an average intensity of 128 and a standard deviation of 20 as this setting could get better accuracy. 7*7 local patch is used for Statistical_Fractal and Binary_Joint. Although large size patch could get better recognition accuracy, it is more time consuming [19] and the main focus of this work is to investigate the effect of binary representation. For comparison, the typical binary feature, LBP, is compared with Statistical_MR8, Statistical_Fractal, Statistical_Joint and the proposed methods. In the following experiments, for LBP, each texture sample was normalized to have an average 128 and a standard of 20 [25]. To get better results, multiscale scheme is used for the LBP method [25]. The Chi-square dissimilarity defined in Section 3.4 and the nearest neighborhood classifier were used for all methods here.

### 1. Experimental Results on CUReT Database

**Figure 10:**
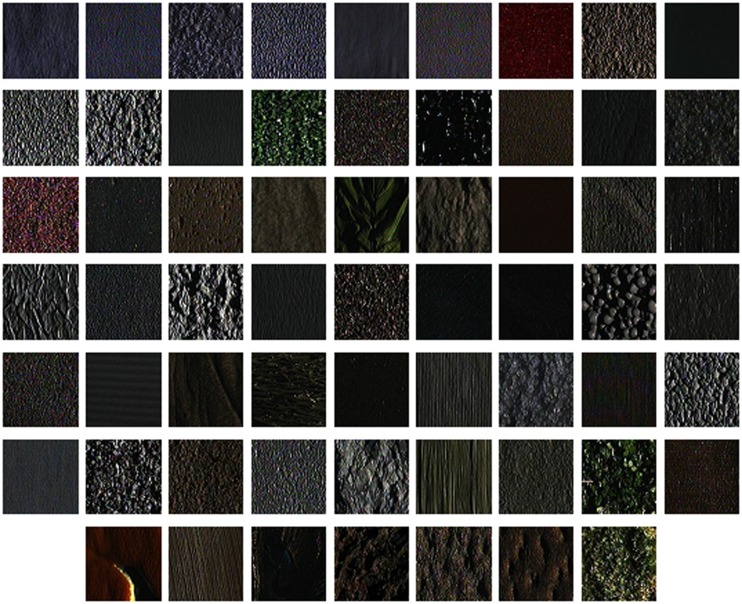
Textures from the Columbia-Utrecht database. In this work, all images are converted to monochrome, so colour is not used to discriminate between different textures.

The CURet database contains 61 textures, as shown in Fig. 10, and there are 205 images of each texture acquired at different viewpoints and illumination orientations. There are 118 images which have been shot from a viewing angle of less than 60°. Of these 118 images, we selected 92 images, from which a sufficiently large region could be cropped (200*200) across all texture classes [17]. To get statistically significant experimental results [19], *L* training images are randomly chosen from each class while the remaining 92-*L* images are used as the test set. The first 23 images of each class are used to learn the library and 40 textons are clustered from each of the texture classes. The average accuracy and standard deviation over 1000 random splits are listed in Table 2.

**Table 2:** Classification rate (%, mean±standard deviation) for CUReT database.

Method	Feature Size	*L*			
		46	23	12	6
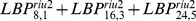	54	95.84±0.82	91.96±1.39	86.41±2.05	78.09±3.33
Statistical_MR8	2440	97.79±0.67	95.03±1.27	90.48±1.98	82.90±3.44
Binary_MR8	2048	98.23±0.58	95.67±1.06	91.11±2.08	83.27±3.75
Statistical_Joint	2440	97.66±0.67	94.58±1.34	89.40±2.39	81.06±3.73
Binary_Joint	2430	96.99±0.65	93.77±1.18	88.49±2.15	80.08±3.53
Statistical_Fractal_D	2440	96.12±0.37*	92.50±0.51*	86.70±0.72*	78.05±0.97*
Statistical_Fractal_L	2440	97.50±0.30*	94.69±0.45*	89.74±0.66*	81.67±0.96*
Binary_Fractal_D	2048	96.20±0.91	92.69±1.39	87.28±2.30	78.98±3.31
Binary_Fractal_L	2048	97.80±0.65	95.14±1.24	90.57±1.98	82.86±3.45

*Classification rates are originally reported in [20]. Other classification rates are computed by us.

Two findings could be found in Table 2. First, the proposed methods are much better than simple LBP method. However, the feature length is a little long. Fortunately, it is not a big issue for nowadays computer.

Second, binary operators show their superiority over statistical operators on CUReT database. For example, Binary_MR8 and Binary_Fractal could get better results than Statistical_MR8 and Statistical_Fractal. While, Binary_Joint could get competitive results with Statistical_Joint.

Furthermore, as there is a training step in statistical operators, the performance will be related with the training set. Table 3 shows the classification rate of Statistical_MR8 and Statistical_Joint under different training samples. Here *C* is the number of classes of training samples to learn the texton dictionary. As shown in Table 3, when the training set is reduced, the performance will be degraded.

**Table 3:** Classification rate (%, mean±standard deviation) for Statistical_MR8 and Statistical_Joint on CUReT database with different training conditions.

Method	*L*			
	46	23	12	6
Statistical_MR8(*C* = 61)	97.79±0.67	95.03±1.27	90.48±1.98	82.90±3.44
Statistical_MR8(*C* = 20)	96.65±0.79	93.45±1.41	88.40±2.12	80.35±3.51
Statistical_MR8(*C* = 10)	95.82±0.87	92.32±1.33	86.92±2.21	78.65±3.50
Statistical_Joint(*C* = 61)	97.66±0.67	94.58±1.34	89.40±2.39	81.06±3.73
Statistical_Joint(*C* = 20)	97.21±0.73	93.83±1.44	88.45±2.42	79.83±3.81
Statistical_Joint(*C* = 10)	96.87±0.74	93.41±1.48	88.00±2.31	79.34±3.86

Classification rates are computed by us.

**Figure 11:**
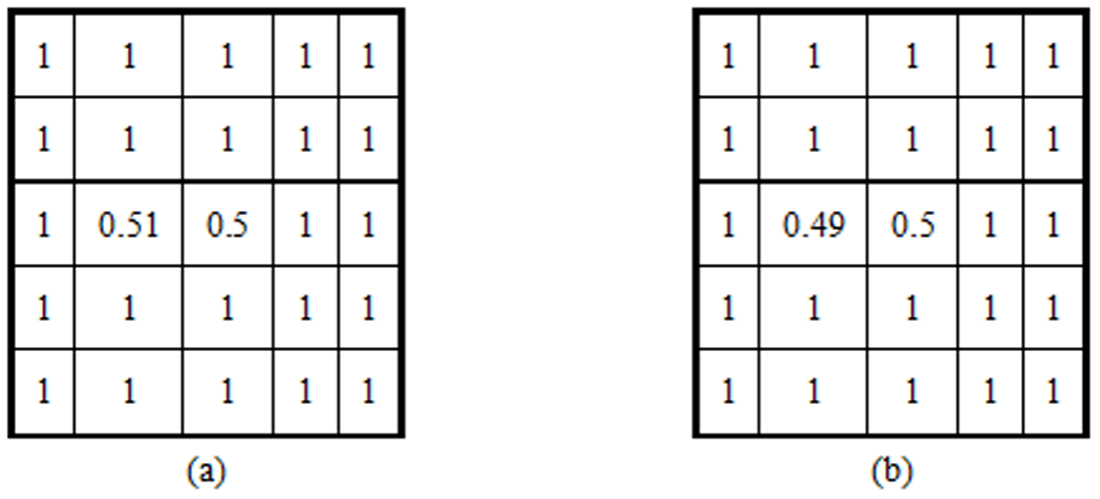
Two 5*5 local patches. These two patches may generate the same statistical texons, but different binary textons.

Compared with statistical texton, binary texton is sensitive to noise. A small disturbance may output the same statistical textons, but the binary textons will be different. Fig. 11 shows an example, in a 5*5 local patch, a pixel value is changed from 0.51 to 0.49, while other pixels are the same. As the difference is very small, the statistical texton may be the same, however, as illustrated in Section 3.3, the binary texton will be significantly different.

To further illustrate noise effect, we created three texture databases with noise:
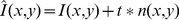
(15)where 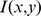 is the gray value of original image pixel and 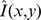 is the transformed value. 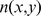
is random noise with 0 mean and 1 standard. *t* (*t* = 1,2,3) is the parameter to control the degree of noise. Table 4 shows the classification rate for different datasets. As shown in Table 4, statistical texton method could get sound results even when *t* = 3, while Binary_Joint fails to over 30% at the same condition. So a preprocessing technique is necessary for binary operators if the image contains severe noise.

**Table 4:** Classification rate (%, mean±standard deviation) for noise sets.

Method	*L*			
	46	23	12	6
Statistical_Joint(*t* = 1)	96.87±0.77	93.20±1.43	87.52±2.47	78.71±3.77
Statistical_Joint(*t* = 2)	91.74±1.00	87.28±1.49	81.11±2.39	72.27±3.61
Statistical_Joint(*t* = 3)	71.99±1.07	68.03±1.25	62.95±1.68	56.34±2.59
Binary_Joint(*t* = 1)	89.78±1.03	85.02±1.44	78.87±2.25	70.38±3.46
Binary_Joint(*t* = 2)	50.71±0.89	48.04±0.91	44.98±1.28	41.14±1.86
Binary_Joint(*t* = 3)	25.04±0.57	23.71±0.59	22.41±0.68	20.87±0.86

Classification rates are computed by us.

### 2. Experimental Results on UIUC Database

**Figure 12:**
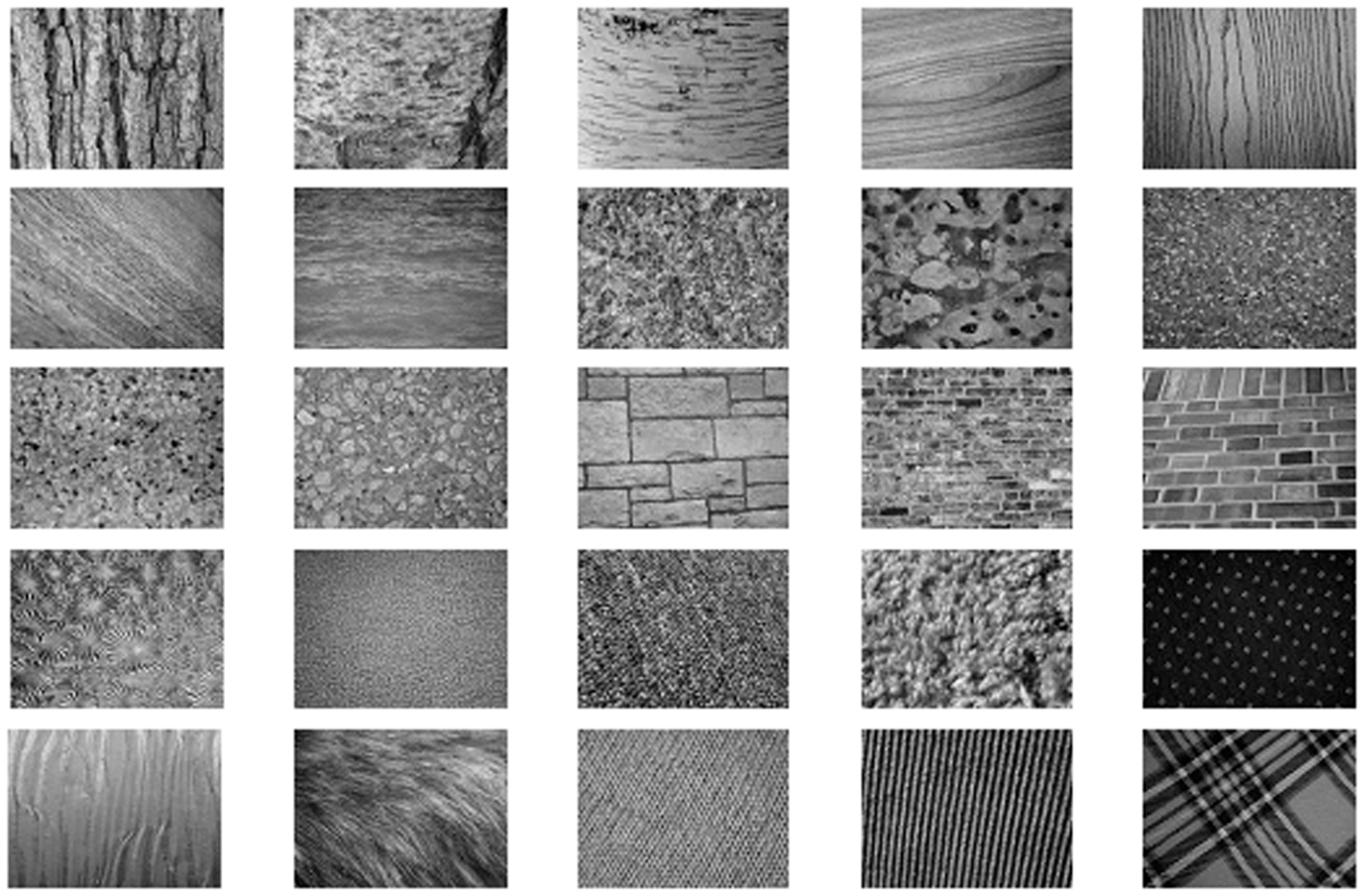
Samples of the 25 textures in UIUC database.

The UIUC texture database [14] includes 25 classes and 40 images in each class. The resolution of each image is 640*480. The database contains materials imaged under significant viewpoint variations as shown in Fig. 12. Similar to Section 4.1, *L* training images are randomly chosen from each class while the remaining 40-*L* images are used as the test set. The first 10 images of each class are used to learn the library and 100 textons are clustered from each of the texture classes. The average accuracy and standard deviation over 1000 random splits are listed in Table 5.

**Table 5:** Classification rate (%, mean±standard deviation) for UIUC database.

Method	Feature Size	*L*			
		20	15	10	5
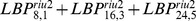	54	76.83±1.91	72.87±1.88	66.68±1.91	55.26±2.11
Statistical_MR8	2440	92.94±1.06∧	91.16±1.11∧	88.29±1.32∧	81.12±1.74∧
Binary_MR8	2048	89.33±1.46	86.53±1.48	81.95±1.72	71.96±2.09
Statistical_Joint	2440	83.54±1.76	80.17±1.81	74.72±1.76	64.26±2.18
Binary_Joint	2430	67.93±2.26	63.04±2.02	56.19±1.97	44.76±1.93
Statistical_Fractal_D	2440	95.40±0.92*	94.09±0.98*	91.64±1.18*	85.35±1.69*
Statistical_Fractal_L	2440	94.96±0.91*	93.66±0.96*	91.25±1.13*	84.96±1.66*
Binary_Fractal_D	2048	85.03+1.63	81.85+1.62	76.56+1.72	65.60+2.13
Binary_Fractal_L	2048	89.17+1.19	86.58+1.32	82.14+1.59	72.25+2.00

*Classification rates are originally reported in [20].

? Classification rates are originally reported in [19]. Other classification rates are computed by us.

Several findings could be found in Table 3. First, similar to what we found in Table 2, the proposed methods are much better than simple binary operators, LBP.

Second, the proposed scheme fails to get better esults than statistical texton methods. This is possibly because of the high resolution. As the image resolution is much higher, statistical textons could describe subtle differences. For example, in UIUC database, there is 4.71% possibility that two different statistical textons have an identical binary texton. While in CUReT database, the possibility is only 2.22%. To further illustrate the resolution effect for statistical and binary operators, we manually down-sampled every image of UIUC database to 1/2 and 1/4 of its original size and applied Statistical_Joint and Binary_Joint methods on these two databases. As shown in Table 6, because statistical textons could describe subtle differences, the classification rate is lower when the image is down-sampled, on the contrary binary texton may get better result. Thus, statistical texton methods are more favorable for high resolution images.

**Table 6:** Classification rate (%, mean±standard deviation) for different resolution.

Method	*L*			
	20	15	10	5
Statistical_Joint (1/2 Image)	81.47±1.66	78.16±1.72	72.64±1.74	62.05±2.02
Statistical_Joint (1/4 Image)	79.32±1.73	75.76±1.64	70.69±1.71	61.14±1.94
Binary_Joint (1/2 Image)	70.40±2.11	65.75±2.04	59.08±1.98	47.99±2.19
Binary_Joint (1/4 Image)	71.74±1.96	67.72±1.97	61.43±1.98	50.44±2.11

Classification rates are computed by us.

### 3. Experimental Results on KTH-TIPS Database

**Figure 13:**
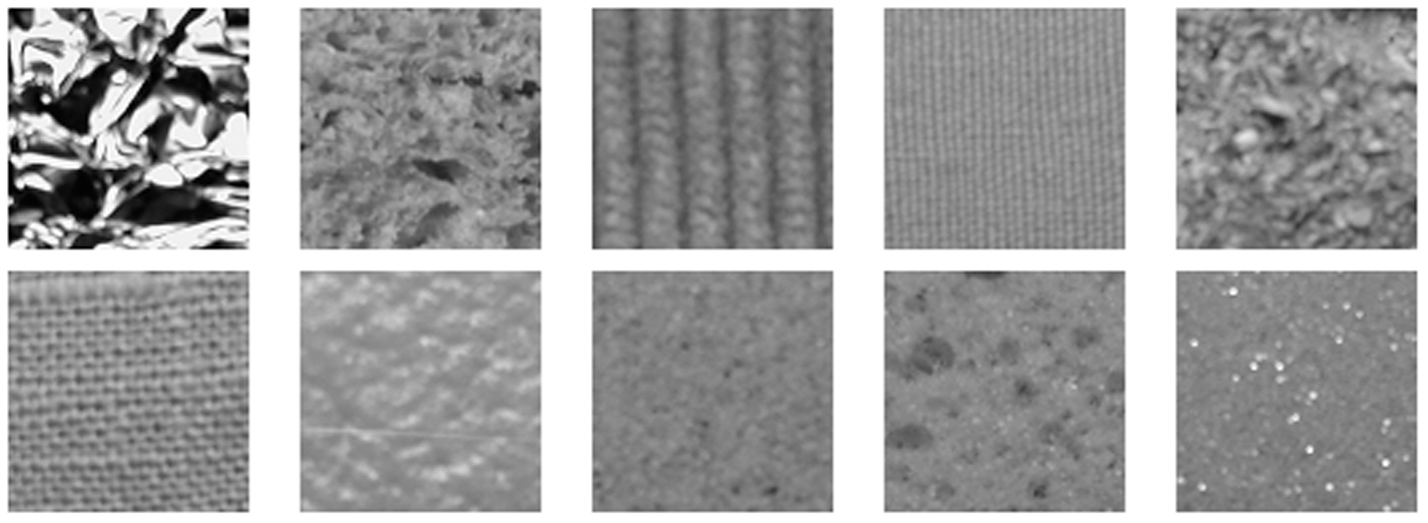
Samples of the 10 textures in KTH-TIPS database.

The KTH-TIPS texture database [28] contains 10 kinds of materials. Images were taken at 9 different scales spanning two octaves. At each scale, 9 images were taken in a combination of three poses and three illumination conditions. Thus, for each class, there are 81 image samples. A 200*200 patch is manually cropped from each sample. However, for some images, due to large camera-target distances, some samples are smaller than 200*200 pixels. Fig. 13 shows image samples of KTH-TIPS database. Similar to Section 4.1, *L* training images are randomly chosen from each class while the remaining 40-*L* images are used as the test set. The first 20 images of each class are used to learn the library and 250 textons are clustered from each of the texture classes. The average accuracy and standard deviation over 1000 random splits are listed in Table 7.

**Table 7:** Classification rate (%, mean±standard deviation) for KTH-TIPS database.

Method	Feature Size	*L*			
		20	15	10	5
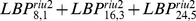	54	95.17±2.22	88.90±3.30	79.18±4.00	67.29±4.97
Statistical_MR8	2500	96.29±1.60	90.46±2.91	81.95±3.67	71.25±5.31
Binary_MR8	2048	96.21±1.28	91.37±2.03	83.72±3.14	73.08±4.91
Statistical_Joint	2500	98.02±1.21	94.40±2.25	88.15±2.73	79.12±4.45
Binary_Joint	2430	95.84±1.82	89.88±2.57	81.05±3.10	70.78±3.99
Statistical_Fractal_D	2500	93.23±1.79	88.66±2.61	82.05±3.40	74.47±4.15
Statistical_Fractal_L	2500	90.49±2.20	83.88±2.99	74.54±3.68	63.32±4.93
Binary_Fractal_D	2048	95.59±1.88	89.57±3.36	81.09±3.74	70.10±4.66
Binary_Fractal_L	2048	94.57±1.89	88.16±3.05	79.07±3.60	68.20±4.71

Classification rates are computed by us.

Similar findings could be found in Table 7. First, the proposed methods are much better than simple LBP method. Second, as the image resolution is not high, binary texton methods could still get good performance. Binary_MR8 and Binary_Fractal could get better results than Statistical_MR8 and Statistical_Fractal, respectively. But, Binary_Joint is a little worse than Statistical_Joint.

### 4. Time Cost

The proposed methods and statistical textons are implemented using Matlab R2008a on a windows XP, T6400 CPU (2.13 GHz) and 2 GB Ram PC. As the feature length are similar for each method and the classifier is the same, we only list the average feature extraction time on different databases. As shown in Table 8, Statistical_Joint method is the most time-consuming method while binary methods are much faster than statistical methods.

**Table 8:** Average time (seconds) cost on feature extraction for different databases.

Method	*Database*		
	CUReT	UIUC	KTH-TIPS
Statistical_MR8	2.3	19.1	1.7
Binary_MR8	1.1	9.0	1.1
Statistical_Joint	26.1	191.1	18.6
Binary_Joint	0.5	4.0	0.5
Statistical_Fractal	5.3	25.3	1.9
Binary_Fractal	1.6	16.1	1.6

## Conclusion

In this paper, we proposed three binary texton methods and reported their experimental results with their statistical counterpart on three large public texture databases. We empirically found that statistical method could get good results for most cases. However, it may be time consuming for feature extraction, especially when the image size is big. Furthermore, it requires a training step to build the texton dictionary which may limit the accuracy when the training sample is not enough. For good quality images with small image size, binary texton methods could get better results than statistical ones. And it does not require training step and is fast for feature extraction. As different schemes have different advantages, future work should investigate how to utilize these properties and improve the classification rate further.
